# CDC Grand Rounds: A Public Health Approach to Prevention of Intimate Partner Violence

**Published:** 2014-01-17

**Authors:** Howard R. Spivak, E. Lynn Jenkins, Kristi VanAudenhove, Debbie Lee, Mim Kelly, John Iskander

**Affiliations:** 1 Div of Violence Prevention, National Center for Injury Prevention and Control, CDC; 2 The Virginia Sexual and Domestic Violence Action Alliance; 3 Futures Without Violence; 4 Office of the Director, CDC

Intimate partner violence (IPV) is a serious, and preventable, public health problem in the United States. IPV can involve physical and sexual violence, threats of physical or sexual violence, and psychological abuse, including stalking (1). It can occur within opposite-sex or same-sex couples and can range from one incident to an ongoing pattern of violence. On average, 24 persons per minute are victims of rape, physical violence, or stalking by an intimate partner in the United States (2). These numbers underestimate the problem because many victims do not report IPV to police, friends, or families. In 2010, IPV contributed to 1,295 deaths, accounting for 10% of all homicides for that year (3). The combined medical, mental health, and lost productivity costs of IPV against women are estimated to exceed $8.3 billion per year (4). In addition to the economic burden of IPV, victims are more likely to experience adverse health outcomes, such as depression, anxiety, posttraumatic stress disorder symptoms, suicidal behavior, sexually transmitted infections, and unintended pregnancy (5).

Among victims of IPV, women are at least three times more likely than men to experience injury from partner violence. Women also are more likely to experience severe physical (24.3%) and sexual violence from a partner, and twice as likely to be killed (2,5). However, in the United States, 13.8% of men also have experienced severe physical violence at some point in their lives (2).

Partner violence often begins at a young age. Based on results from the 2011 Youth Risk Behavior Survey, approximately 9% of high school students reported date-related physical violence by a boyfriend or girlfriend (6). Among females who experienced rape, physical violence, or stalking by an intimate partner, 22.4% experienced some form of IPV for the first time at age 11–17 years, 47.1% at age 18–24 years, and 21.1% at age 25–34 years. Among males who experienced rape, physical violence, or stalking by an intimate partner, 15.0% experienced some form of IPV for the first time at age 11–17 year, 38.6% at age 18–24 years, and 30.6% at age 25–34 years (Figure) (2). Many persons who experience IPV while young continue to encounter a pattern of abuse well into adulthood.

The causes of IPV are complex and often the product of multiple individual, relationship, community, and societal factors. Such factors include engaging in aggressive or delinquent behavior as a youth, heavy alcohol or drug use, witnessing or experiencing violence as a child, marital conflict, dominance and control in a relationship, and unemployment (7). Much less is known about community and societal risk factors for IPV, such as high rates of poverty and cultural and social norms that support violence (8).

## Importance of Surveillance

Data collected and interpreted through public health surveillance support efforts to prevent IPV. CDC uses the National Intimate Partner and Sexual Violence Survey (NISVS)* to collect information on nonfatal IPV. The data are used to identify populations at risk, inform prevention efforts, monitor the problems, and assess trends over time. NISVS is the first system to provide national and state data on IPV, sexual violence, and stalking to guide prevention.

CDC also operates the National Violent Death Reporting System (NVDRS).† This is a state-based surveillance system that collects information from various sources about violent deaths, including IPV-related homicides. The information is collected from death certificates, police reports, and coroner/medical examiner reports and stored in an encrypted database. Currently, NVDRS operates in 18 states, consolidating data on violent deaths, unintentional firearm deaths, and deaths of undetermined intent. State and local violence prevention practitioners use these data to guide their prevention programs, policies, and practices. The data also are used to understand the magnitude, trends, and characteristics of violent deaths, and to help evaluate state and local prevention programs and strategies.

## A Public Health Approach to Prevention

Public health has a role in building capacity and expertise within communities to develop and implement evidence-based IPV prevention strategies that target known risk factors. These infrastructure-building efforts can work to identify programs, practices, and policies that moderate or reduce IPV risks, facilitate the scale-up of effective primary and secondary prevention strategies, and ensure wide-spread adoption of those strategies.

Of those strategies that have been evaluated, some are effective in changing knowledge and attitudes, but not actual behaviors (9), and a small but growing number have been shown to reduce partner violence and/or victimization (10,11). Current strategies include youth and parent-focused programs, therapeutic approaches with at-risk couples, community-based programs, and economic and policy-focused approaches. Most of the programs that effectively change behavior target adolescents and prevention of dating violence (10,11). Less is known about effective prevention approaches with adult populations, although some programs with adults have shown promise (12,13). Helping teens learn to establish healthy, nonviolent relationships might reduce the prevalence of adult partner violence over time. The evidence base for effective prevention of intimate partner violence is growing and evolving, and new strategies are being implemented and evaluated (14).

CDC’s Dating Matters§ project is testing strategies that build on what is known about the prevention of teen dating violence. Developed for youth in high-risk urban communities, Dating Matters promotes healthy relationships and prevention of dating violence by combining a variety of prevention strategies that engage youth, their parents, and educators (15,16). In addition, communities assess and inform local policy to support efforts to foster safe and healthy relationships for youth and sustain evidence-based prevention programs. CDC is currently supporting the implementation and evaluation of Dating Matters in four urban communities before disseminating the prevention strategies more widely.

The Family Violence Prevention Services Act (FVPSA), reauthorized in 2010 as part of the Child Abuse Prevention and Treatment Act,¶ gives CDC the authority to invest federal funds to support coordinated community responses to address partner violence. Using FVPSA funds, CDC supported the Domestic Prevention Enhancements and Leadership Through Alliances (DELTA) program,** with a focus on primary prevention of IPV. Through DELTA, CDC funded 14 state domestic violence coalitions (SDVCs) to engage local partners in data-driven planning, prevention-focused training and technical assistance, and state and local support for prevention efforts. These efforts are geared toward identifying, implementing, and evaluating primary IPV prevention strategies.

In 2013, CDC launched DELTA Focusing on Outcomes for Communities United with States (DELTA FOCUS),†† which funds 10 SDVCs. DELTA FOCUS grantees support IPV prevention at the national, state, and local levels through strategies that address the structural determinants of health at the outer layers (societal and community) of the social-ecological model of public health.§§ This means, in addition to addressing individual and relationship factors associated with IPV outcomes, grantees support work to change the environments and conditions in which people live, work, and play. To do this, economic and social policies and processes and norms that shape the health of individuals and communities must be addressed. This might involve strategies that integrate issues related to education, employment, social norms, gender equality, and more.

One IPV prevention effort that has focused on teen dating violence is the Start Strong: Building Healthy Teen Relationships initiative,¶¶ funded by the Robert Wood Johnson Foundation in partnership with Blue Shield of California Foundation. Start Strong was begun in 2008 as a 4-year initiative and has been the largest private sector investment in teen dating violence prevention so far. The initiative identified innovative yet practical solutions to prevent teen dating violence and promote healthy relationships among persons aged 11–14 years in 11 communities. Using the social-ecological model of public health, Start Strong includes strategies to educate and engage youths in and out of the school setting, and to educate and engage teen influencers (e.g., parents, caregivers, older teens, teachers, and other school personnel). Strategies to improve outcomes through increased awareness and behavioral change also rely on coordinated improvements in school district polices promoting prevention and response and using creative social marketing and social media efforts focused on youths and parents.

In 1993, Futures Without Violence was established as the Department of Health and Human Services National Health Resource Center on Domestic Violence.*** Beginning with 12 emergency departments across the United States, this initiative created the first organized opportunity for doctors, nurses, social workers, domestic violence prevention advocates, and police to join forces as equal partners to address IPV. It has since been expanded into five multistate initiatives in various health and public health programs. The focus is to build consensus around recommended violence intervention practices among health-care and public health leaders by understanding what can be done and what changes to the existing health-care systems are necessary. This effort has resulted in improvements to professional training curricula; changes to medical records, charting, and coding techniques; community partnerships; policy improvements; and leadership development. Futures Without Violence is also building on opportunities created by the Affordable Care Act.††† Those include reimbursement for screening and counseling for IPV to facilitate recommendations made by the U.S. Preventive Services Task Force and other organizations to integrate IPV screening, assessment, counseling, and referral into teen pregnancy prevention and other adolescent and reproductive health programs, well-women visits, and home visitation programs.

## The Future of IPV Prevention

Raising awareness and developing rigorous evidence-based programs, practices, and policies to prevent IPV are essential to stopping violent behavior before it starts. Efforts to effectively prevent the start of IPV also need to focus on healthy relationships across the lifespan, with a particular emphasis on children and youth. Early education and prevention provide the best hope for creating healthy futures and fostering a society without domestic violence.

More research on longitudinal risk for IPV and protective factors is needed to better understand what works, and rigorous evaluation of prevention strategies that are being implemented is critical. Programs, practices, and policies need to be developed that are culturally based and responsive to the populations at greatest risk, and evidence needs to be gathered on how best to scale-up effective approaches to ensure widespread adoption.

Given the social and environmental complexities of IPV, collaborators within and outside public health need to be involved in finding solutions. The problem of IPV can only be addressed if the focus is shifted from responding to acts of violence to preventing violence before it starts. This will require the involvement of many key sectors, including education, the media, housing and community development, criminal justice, transportation, and private industry. Public health entities and SDVCs have a history of being effective champions of multidisciplinary and multisector initiatives (17,18). Ultimately, rigorous evaluation of the outcomes of prevention efforts makes it possible to determine the long-term impact on population health, inform policy decisions, and build effective strategies to prevent IPV.

## Figures and Tables

**FIGURE:**
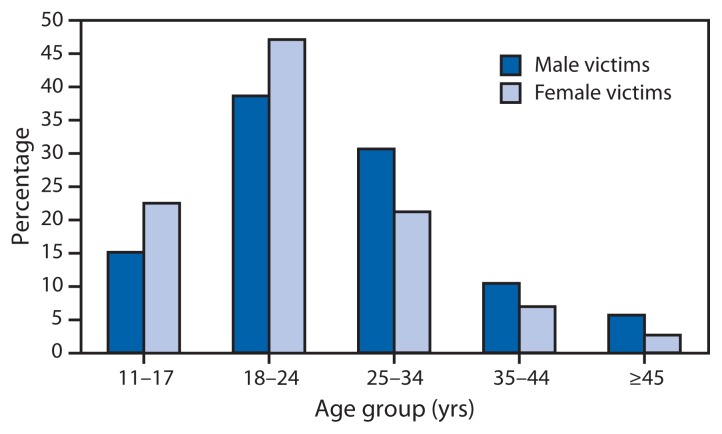
Age at occurrence of first intimate partner violence experience among males and females who experienced rape, physical violence, or stalking by an intimate partner — National Intimate Partner and Sexual Violence Survey, United States, 2010

## Footnotes

* Additional information available at http://www.cdc.gov/violenceprevention/nisvs.

† Additional information available at http://www.cdc.gov/violenceprevention/nvdrs.

§ Additional information available at http://www.cdc.gov/violenceprevention/datingmatters.

¶ Child Abuse Prevention and Treatment Act, Pub. L. No. 111-320, 124 Stat. 3459 (December 20, 2010). Available at http://www.gpo.gov/fdsys/pkg/PLAW-111publ320/pdf/PLAW-111publ320.pdf.

** Additional information available at http://www.cdc.gov/violenceprevention/delta.

†† Additional information available at http://wwwdev.cdc.gov/violenceprevention/deltafocus.

§§ Additional information available at http://www.cdc.gov/violenceprevention/overview/social-ecologicalmodel.html.

¶¶ Available at http://startstrong.futureswithoutviolence.org/learn-more.

*** Additional information available at http://www.futureswithoutviolence.org/content/features/detail/790.

††† Patient Protection and Affordable Care Act of 2010. Pub. L. No. 114-48 (March 23, 2010), as amended through May 1, 2010. Available at http://www.healthcare.gov/law/full/index.html.
